# Ingredient-dependent water mobility and physicochemical properties of model tomato ketchup in relation to serum separation

**DOI:** 10.1007/s10068-025-01947-3

**Published:** 2025-07-16

**Authors:** Choon-Gil Kang, Hyejeong Shin, Shinjae Park, Shin-Joung Rho, Yong-Ro Kim

**Affiliations:** 1 Department of Biosystems Engineering, Seoul National University, Seoul, 08826 Republic of Korea; 2 Ottogi Corporation, Anyang, Gyeonggi-Do 14060 Republic of Korea; 3 Center for Food and Bioconvergence, Seoul National University, Seoul, 08826 Republic of Korea; 4 Research Institute of Agriculture and Life Sciences, Seoul National University, Seoul, 08826 Republic of Korea

**Keywords:** Tomato ketchup, Serum separation, LF-NMR relaxation time, Water mobility, Rheological property, Regression analysis

## Abstract

This study was performed to evaluate the effects of added ingredients such as modified starch (MS), xanthan gum (XG), sugar, (SU), salt (SA), and vinegar (VIN) on the water mobility and physicochemical properties of model tomato ketchup and to investigate the correlation between the obtained variables and serum separation. The type and concentration of ingredients added to the tomato paste (TP) had significant effects on experimental variables, including the serum separation rate, water mobility, and viscosity. Serum separation was most severe in SU and VIN- added samples and minimal in MS and XG- added samples. Principal component analysis exhibited that several physical variables (T2 relaxation time, flow behavior properties, storage and loss modulus) were related to serum separation. The causes of these separation events may vary depending on the specific properties of the added ingredients.

**Supplementary Information:**

The online version contains supplementary material available at 10.1007/s10068-025-01947-3.

## Introduction

Tomato is one of the most important vegetables worldwide and processed to various products, including TP, tomato juice, tomato ketchup, various sauces etc. Tomato ketchup is one of most popular processed tomato products made of TP with ingredients such as sugar, vinegar, salt and various spices (Juszczak et al., 2013). The quality of tomato ketchup can be affected by its ingredients. Salt can improve the color stability by acting as an inhibitor of the browning enzyme. Sugar and vinegar prevent the growth of microorganisms to prevent spoilage. Starch and hydrocolloids are used as thickeners and stabilizers. Various hydrocolloids (carboxy methylcellulose, xanthan gum, modified starch, etc.) also affect the consistency of tomato ketchup by increasing its viscosity and interact with solids and water to improve physical stability (Koocheki et al., 2009; Sahin and Ozdemir, 2004, 2007). Therefore, the monitoring of quality parameters, such as rheological properties, water mobility, and the appearance index (such as serum separation and color uniformity) with the addition of various ingredients is important to maintain and improve the quality of tomato ketchup.

Serum separation is one of the most important quality factors to consider in processing sauce products, especially when applying a new ingredient or formulation. Extensive studies have been conducted to analyze various factors affecting serum separation of tomato ketchup, such as the degree of homogenization, solid content, and viscosity with the addition of gums and MS (Komeilyfard et al., 2017; Sahin and Ozdemir, 2007; Stoforos and Reid, 1990). Serum separation in foods with relatively high solid contents, such as tomato ketchup, sauces, and pastes, is influenced by various factors, including the amount of insoluble solids, the types and concentrations of soluble ingredients (such as salts, sugars, gums, starches, and gelatin), the degree of homogenization, and whether or not heating is applied. However, conventional methods for measuring serum separation, such as visual inspection or centrifugation, are not only time-consuming but also limited in accuracy and objectivity. These approaches often lack sensitivity to detect early or subtle phase separation. Therefore, they are not ideal for systematically evaluating the effects of different ingredients on the physical stability of complex food systems like tomato ketchup.

Low-field nuclear magnetic resonance (LF-NMR) offers an effective approach for detecting changes in water mobility within complex food systems. The water mobility within food matrices can be directly measured by analyzing the relaxation time in the magnetic field generated by hydrogen nuclear spins in water. Due to the usefulness of LF-NMR, it has been used to measure ingredient quality, monitor processes, and predict the storage stability of various foods. Among them, the technologies of solid fat content measurement and monitoring the freezing process or fat hardening process are actually used in the food industry (Capitani et al., 2017). LF-NMR has been widely applied to evaluate the water mobility of different food materials, particularly bread, milk, pork and soybean protein (Guo et al., 2014; Li et al., 2013; Lin et al., 2016; Salomonsen et al., 2007). Also, various researches have been conducted using LF-NMR to monitor the spoilage of TP (Pinter et al., 2014) and to evaluate the effects of ingredients such as flour, gelatin and salt and various hydrocolloids on the characteristics of tomato sauce (Carini et al., 2015; Diantom et al., 2017). Diantom et al. (2017) reported that changes in the physical properties of tomato sauce with the addition of gums, proteins, and dietary fibers were related to the proton T_2_ relaxation time measured by LF-NMR. However, despite the widespread use of LF-NMR to analyze water mobility in food, the stability of ketchup, particularly serum separation, has been assessed only through macroscopic approaches (Mirzaei et al., 2018; Omidbakhsh amiri et al., 2015; Stoforos and Reid, 1992), highlighting the need for NMR-based investigations in this area. The behavior of water greatly affects the quality and storage stability of foods, including those with high solids content such as ketchup, influencing the phase separation rate and rheological properties. These properties are all related to the water mobility interacting with the various components of ketchup. Water molecules in food are physically trapped in the food matrix or bound through chemical interactions with food components such as carbohydrates and proteins and thus exhibit various molecular mobility levels depending on the environment around the water molecules. Therefore, it is necessary to analyze and understand the movement of water molecules in food, as their behavior plays an important role in determining the physical properties, functionality, and stability of food. The behavior of water molecules in tomato ketchup is influenced not only by the overall formulation but also by the specific types and concentrations of ingredients, indicating an “ingredient-dependent” effect on physical stability and water mobility. Moreover, it seems possible to elucidate the effects of ingredient types and concentrations on the physical properties and stability (serum separation) of tomato ketchup through extensive analysis of water mobility.

In this study, effects of conventional tomato ketchup ingredients on various physicochemical properties (water mobility, viscoelastic behavior, flow behavior) and serum separation of model tomato ketchup were investigated. To analyze the relationship between those physicochemical properties and serum separation and the effect of ingredients, principal component analysis (PCA) was conducted. This study can provide the insight to better understand the effect of ingredients on the notorious problem of tomato ketchup, serum separation, in tomato product development.

## Materials and methods

### Materials

Tomato paste (31°Brix, The Morning Star Packing Co., Los Banos, Calif., USA)(TP), chemically modified starch (acetylated di-starch adipate from waxy corn starch, Roquetteq S.P.A., Cassano Spinola, Italy)(MS), xanthan gum(Jungbunzlauer S.A.S., Marckolsheim, France)(XG), sugar (TS Corporation, Incheon, Republic of Korea)(SU), refined salt (Hanjusalt, Ulsan, Republic of Korea)(SA), and vinegar (OTTOGI Co., Ltd. (Anyang, Republic of Korea)(VIN).

### Preparation of model tomato ketchup

In order to investigate the physicochemical property changes of TP depending on the supplemented ingredient type and concentration used in the tomato ketchup production, each ingredient was separately mixed with TP to make model tomato ketchup at different concentrations, the ranges of which were similar to those used in conventional ketchup production (Porretta, 1991) as shown in Table 1. For all samples, the ratio of TP to water was 1:1, and the control TP was a simple mixture of TP and water. In order to prepare MS and XG, MS (10%, w/v) was gelatinized at 80 °C for 10 min and XG was dispersed in water before mixing. All samples were supplemented with 0.05wt% sodium benzoate as a preservative and stored at 4 °C for 24 h prior to analyses except for serum separation measurement.

**Table 1:** Model tomato ketchup formulation (wt %)

Samples	Conc. (wt%)	Tomato paste	Water	Modified Starch	Xanthan gum	Sugar	Salt	Vinegar
Tomato paste		50	50	0	0	0	0	0
Modified starch	0.5	49.75	49.75	0.5	0	0	0	0
	1	49.5	49.5	1	0	0	0	0
	1.5	49.25	49.25	1.5	0	0	0	0
Xanthan gum	0.05	49.975	49.975	0	0.05	0	0	0
	0.1	49.95	49.95	0	0.1	0	0	0
	0.15	49.925	49.925	0	0.15	0	0	0
Sugar	10	45	45	0	0	10	0	0
	20	40	40	0	0	20	0	0
	30	35	35	0	0	30	0	0
Salt	1.5	49.25	49.25	0	0	0	1.5	0
	3	48.5	48.5	0	0	0	3	0
	4.5	47.75	47.75	0	0	0	4.5	0
Vinegar	4	48	48	0	0	0	0	4
	8	46	46	0	0	0	0	8
	12	44	44	0	0	0	0	12

### Serum separation

Serum separation of each mixed sample was determined by screen tube method according to (Stoforos and Reid, 1990) with some modifications. A screen tube was fabricated by attaching a 60 mesh/inch stainless steel screen to an acrylic tube with a height of 10 cm, inner diameter of 3.2 cm, and outer tube diameter of 3.6 cm. The weight of outer tube was measured prior to the experiment. After each sample (5–5.5 g, w/v) was placed on the screen, tubes were wrapped, completely sealed with parafilm, the serum drainage through the screen was measured at different time points during 6 weeks. The serum separation rate was calculated as the amount of separated serum relative to the initial sample weight as seen in Eq. 1:

1 $${\text{Serum separation rate }}\left( \% \right) = \frac{{{\text{Serum weight }}\left( {\text{g}} \right)}}{{{\text{Initial sample weight }}\left( {\text{g}} \right)}} \times 100$$

The final serum separation rate was expressed as drip (%). In order to evaluate serum separation kinetics, the serum retention rate (1 – serum separation rate/100) data were fitted to Avrami’s equation (Yoshii et al., 2001) (Eq. 2):

2 $${\text{R }} = \, \exp \, \left( { - {\text{kt}}^{{\text{n}}} } \right)$$

where R is a serum retention rate, *k* (h^−1^) is a serum separation rate constant, and* n* is a parameter representing the release mechanism.

### Water content

The water content (WC, %) of the tomato sample was measured by the difference in weight before and after drying at 90 °C in an infrared moisture meter (FD-600, Kett electric laboratory Co., Ltd, Tokyo, Japan).

### ***Low-field***.^***1***^***H nuclear magnetic resonance (LF-NMR)***

Water mobility was analyzed using a low-field (20 MHz) ^1^H nuclear magnetic resonance (LF-NMR) spectrometer (the MiniSpec, Bruker Biospin, Milano, Italy) set at 25 °C with a 400L/h gas flow. The tomato samples were filled to a height of about 7 cm in a 10 mm diameter NMR tube and sealed with parafilm to prevent moisture loss. T_2_ (spin–spin) relaxation time was measured using Carr-Purcell-Meiboom-Gill (CPMG) pulse sequence (τ − 180° − τ)_n_. The optimal conditions for the T_2_ relaxation time measurement variables of the samples obtained through preliminary experiments were as follows: a 90° Pulse length of 3 μs, a tau of 1 ms, a recycle delay of 2 s, a gain (Db) of 68, 1024 data points, and 16 or 32 scans. The measured data were fitted with one and two exponential component models, respectively, using LF-NMR Toolbox (Pedersen et al., 2002) of Matlab (The Mathworks Inc. Natric, MA) (Eq. 3 and 4):

3 $$f\left( {\text{t}} \right) \, = {\text{ offset }} + {\text{ A }} \times {\text{ exp}}\left( { - {\text{t}}/{\text{T}}_{{{\text{2A}}}} } \right)$$

4 $$f\left( {\text{t}} \right) \, = {\text{ offset }} + {\text{ a }} \times {\text{ exp}}\left( { - {\text{t}}/{\text{T}}_{{{\text{2a}}}} } \right) \, + {\text{ b }} \times {\text{ exp}}\left( { - {\text{t}}/{\text{T}}_{{{\text{2b}}}} } \right)$$

where T_2A_ (ms) is the relaxation time of the one component model and T_2a_ and T_2b_ (ms) are the relaxation times (T_2a_ < T_2b_) of two components model. The variables A, a, and b (%) were expressed as I_A_, I_a_, and I_b_, respectively, as the relative signal intensities of each model representing the water population (area ratio) corresponding to each T_2_ component.

### Rheological measurements

Rheological properties of tomato samples were carried out with a rheometer (AR 1500ex, TA Instruments Ltd., New Castle, DE, USA), using a parallel plate geometry (40 mm of diameter). Samples were loaded in the bottom plate and pressed down with the top plate to have 1 mm gap between them. Excess sample was removed from the edge of plates and then covered with a thin layer of low-density silicon oil to prevent evaporation loss.

To evaluate the viscoelastic properties, the storage modulus (G', Pa) and loss modulus (G”, Pa) were measured using a frequency sweep test (0.5% strain, 25℃, 0.1–10 Hz). The moduli data against frequency (*f*) were fitted to power functions as below (Eq. 5 and 6).

5 $$G\prime \, = K\prime \left( f \right)^{{n^{\prime}}}$$

6 $$G^{\prime\prime} \, = K^{\prime\prime}\left( f \right)^{{n^{\prime\prime}}}$$

The equation variables were experimentally determined constants, where *K'* (Pa·s^n’^) and *K''* (Pa·s^n’’^) represent G' and G'' at 1 Hz frequency, and n' and n'' represent the slopes of G' and G'' for frequency change.

The steady flow tests were performed at 25℃ in the shear rate (γ) range of 0.1 – 500 s^−1^, and shear stress-shear rate curves were fitted to the Herschel–Bulkley model (Eq. 7).

7 $$\tau \, = \tau_{0} + K\gamma^{n}$$

where τ is a shear stress (Pa), τ_0_ is a yield stress (Pa), γ is a shear rate (s^−1^), *K* is a consistency coefficient (Pa·s^n^), and *n* is a flow behavior index. In addition, the apparent viscosity at shear rate of 10 s^−1^ (η_10_) was also calculated by dividing the shear stress (τ) with the shear rate (γ) at 10 s^−1^.

### Bostwick consistency

Tomato sample consistency was measured as a flow distance in a Bostwick consistometer (CSC Scientific Co., Inc., Fairfax, VA, USA). The Bostwick consistometer chamber was filled with a sample equilibrated at 20 °C and then the flow distance (mm) of the sample was measured for 30 s.

### Statistical analysis

All experiments were conducted in two or three replicates. The one-way analysis of variance (ANOVA) was used to identify the effect of each ingredient and concentration with TP. The significant differences among the mean values were estimated using Tukey’s multiple comparison test (*p* < 0.05). In addition, principal component analysis (PCA) were performed to understand the correlation between variables and to explain the relationship by downsizing the variables. All experimental data was statistically analyzed using SPSS version 25.0 (SPSS Inc., Chicago, IL, USA) and PCA analysis was performed using Python with necessary Application Programing Interfaces (API) in Scikit-Learn library running in Google Colab.

## Results and discussion

### Effects of ingredients on serum separation

**Fig. 1:**
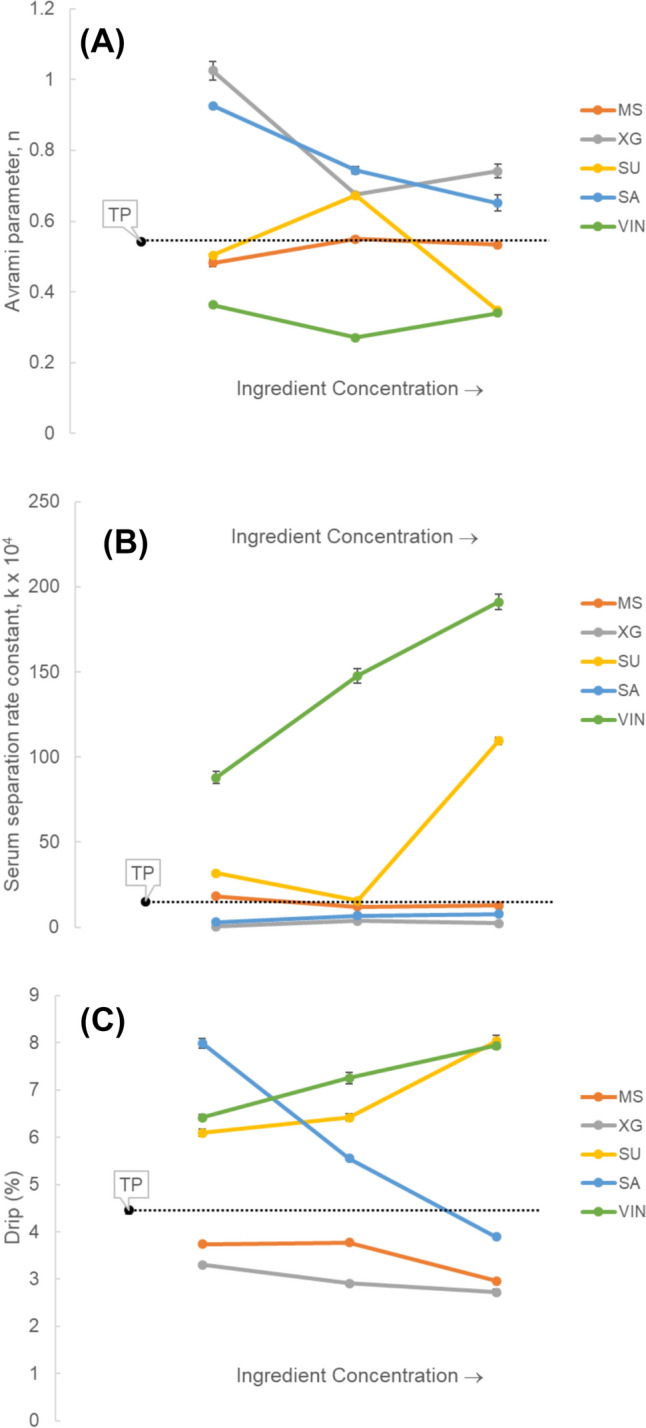
**(A)** Avrami parameter, n, **(B)** serum separation constant, k, and **(C)** drip (%) estimated from the data of serum retention rate measured during 6 weeks of storage for model tomato ketchup samples prepared with different ingredients and concentrations. Three different ingredient concentrations used to make each model tomato ketchup are as follows. MS – 0.5, 1, and 1.5% modified starch; XG – 0.05, 0.1, and 0.15% xanthan gum; SU – 10, 20, and 30% sugar; SA – 1.5, 3, and 4.5% salt; VIN – 4, 8, and 12% vinegar. TP is the control tomato paste

Tomato ketchup is a representative tomato product based on TP with different additives or ingredients typically including MS, gums (typically XG), SU, SA, and VIN (Porretta, 1991). To investigate the effects of each ingredient on tomato ketchup stability, model tomato ketchup samples containing TP and each ingredient were prepared and their serum retention rates (R) were measured during 6 weeks of storage (Fig. S1). Regardless of added ingredients, serum separation rapidly occurs during the initial 2–3 weeks and then slowed down. The initial fast decrease of serum retention rate was fitted to Avrami’s equation (Eq. 2) to estimate the Avrami parameter, *n* and the serum separation rate constant *k* (Fig. 1). All the values of *n*, representing the release mechanism, were less than 1 except for XG 0.05 (TP + 0.05% xanthan gum), indicating that the molecular diffusion rate was a limiting factor and there was no obvious induction period before serum separation (Yoshii et al., 2001). Among samples, VIN showed the lowest values of *n* and the highest values of *k*, indicating that the initial serum separation was most pronounced with VIN addition in a concentration dependent manner. This phenomenon can be attributed to both the high water content of VIN, which raises the proportion of free water in the system, and its acidic nature. The decrease in pH may reduce the electrostatic repulsion among biopolymers, leading to protein aggregation and destabilization of the matrix (Hu et al., 2019; Igartúa et al., 2022). SU also showed higher *k* than that of TP, especially at high sugar concentration (30%). This is likely due to osmotic pressure effects, wherein SU draws water out of the TP matrix, increasing the level of free water and leading to visible serum separation (Nieto et al., 2013). In contrast, MS, XG, and SA showed similar or lower *k* values compared to TP.

Among all the model tomato ketchup samples, XG showed the lowest drip for all XG concentrations (0.05, 0.1, and 0.15%). VIN and SU at high concentrations (12 and 30%, respectively) and SA at low salt concentration (1.5%) showed the highest drip. Interestingly, a small amount of added SA resulted in a high level of serum separation, likely due to osmotic effects. However, at higher SA concentrations, serum separation decreased sharply and appeared to have little to no impact. One possible explanation is that SA interacted with the native components of TP, such as proteins and dietary fibers, thereby enhancing water retention and structural stability. Ionic interactions may have reduced electrostatic repulsion between these components, promoting the formation of a more rigid matrix and minimizing serum separation (Pastorino et al., 2003). In addition, the suspended tomato solids, which occupy the largest volume in each sample, contribute to system stability through interparticle interactions (Mizrahi, 2010). However, high concentration of SU and VIN could interfere with these interactions, leading to increased syneresis. The effects of hydrocolloids, which decreased drip, could be due to an increase in water holding capacity resulting from interactions between water and hydrocolloid molecules (Omidbakhsh amiri et al., 2015).

### Effects of ingredients on water mobility

#### Low-field ^1^H nuclear magnetic resonance

**Fig. 2:**
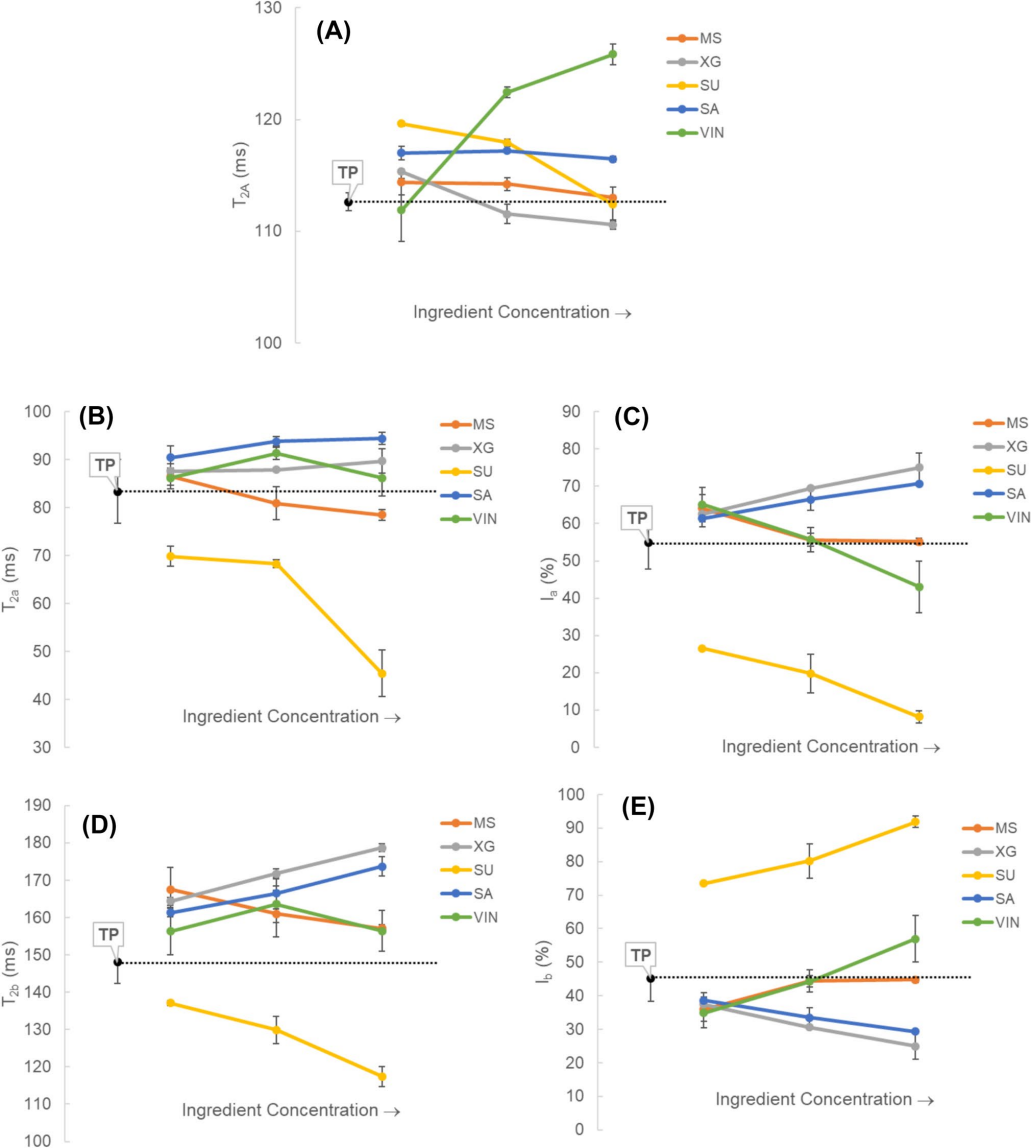
**(A)** One exponential T_2_ relaxation times (T_2A_), **(B)** fast components of two exponential T_2_ relaxation times (T_2a_) and **(C)** their signal intensities (I_a_), **(D)** slow components of two exponential T_2_ relaxation times (T_2b_) and **(E)** their signal intensities (I_b_) of model tomato ketchup samples prepared with different ingredients and concentrations. Three different ingredient concentrations used to make each model tomato ketchup are as follows. MS – 0.5, 1, and 1.5% modified starch; XG – 0.05, 0.1, and 0.15% xanthan gum; SU – 10, 20, and 30% sugar; SA – 1.5, 3, and 4.5% salt; VIN – 4, 8, and 12% vinegar. TP is the control tomato paste

The T_2_ (spin–spin) relaxation time and corresponding relative intensity obtained from LF-NMR measurements were used to explain the relative mobility of water molecules in model tomato ketchup samples with different added ingredients. The T_2_ relaxation parameters obtained from one- and two-exponential models (Eq. 3 and 4, respectively) are shown in Fig. 2. Figure 2a demonstrate the T_2A_ represents average T_2_ relaxation time obtained from mono-exponential data fitting, while T_2a_ and T_2b_ correspond to the relaxation times of immobilized (bound) and free water, respectively, based on bi-exponential modeling. This was assumed as the less and more mobility of each pool when explained by dividing the water mobility in the sample into two characters, where the distribution intensity of the water molecule character with mobility corresponding to T_2a_ and T_2b_ represented I_a_ and I_b_, respectively. In addition, T_2A_ represents the mobility of water molecules as mono character (Salomonsen et al., 2007). These two modelings approach were used in both ways to observe the water mobility once in two sectors, where the sample mobility properties were large and small and once in the whole sample itself.

As seen in Fig. 2c, the I_a_ increased in the order of SA and XG (approx. 55%–68%), and decreased with the addition of SU (approx. 6%–22%). The addition of MS and VIN did not lead to significant differences compared to TP. In the case of I_b_, the intensity of relatively high mobility increased with the addition of SU (approx. 60%–73%) and decreased in the order of XG, and SA (22%–45%), exhibiting the opposite trend to I_a_. The addition of SU induced decreases in T_2a_ (42–70 ms) and T_2b_ (117–138 ms), in contrast to the other ingredients. That is, the addition of MS, VIN, SA, and XG increased the overall molecular mobility.

XG induced a reorganization of water within its polymer network, evidenced by a reduction in total free water alongside an increase in relaxation time for the remaining water molecules. This suggests that water was loosely entrapped, restrained enough to prevent migration but still retaining limited mobility, thereby boosting water holding capacity and reducing serum separation. These observations agree with Diantom et al. (2017), who also reported enhanced proton mobility in XG systems. Conversely, the addition of SU resulted in relatively low serum stability, as evidenced by reductions in both T_2a_ and T_2b_ values, along with a decline in I_a_, indicating weakened interactions among structured water molecules. Meanwhile, an increase in I_b_ was observed, which may reflect not only enhanced mobility of free water protons but also contributions from protons originating from the high concentration of SU. These combined effects suggest a destabilized and heterogeneous environment with increased molecular mobility and less effective water retention within the matrix, ultimately resulting in increased serum separation. The addition of VIN and SA resulted in contrasting effects on water proton mobility and distribution. VIN addition led to increases in T_2A_, T_2a_, T_2b_, and I_b_, while I_a_ decreased. These changes are attributed to the high water content of vinegar, which introduced more free water into the system, thereby enhancing molecular mobility. The reduction in I_a_ indicates a decline in bound water, likely due to dilution of water–polymer interactions. In contrast, SA addition increased T_2A_, T_2a_, T_2b_, and I_a_, but decreased I_b_. This pattern suggests that SA ions facilitated the structuring of water molecules around ions and within the matrix, increasing the proportion of structured or bound water. Meanwhile, the decrease in I_b_ indicates restricted proton mobility, possibly due to electrostatic interactions or proton confinement within hydration shells (Rezaei‐Ghaleh, 2022). Overall, VIN and SA exert opposite effects on the water state: VIN increases the proportion and mobility of free water, while SA promotes water structuring and suppresses proton dynamics.

### Relationship between serum separation and water mobility

**Fig. 3:**
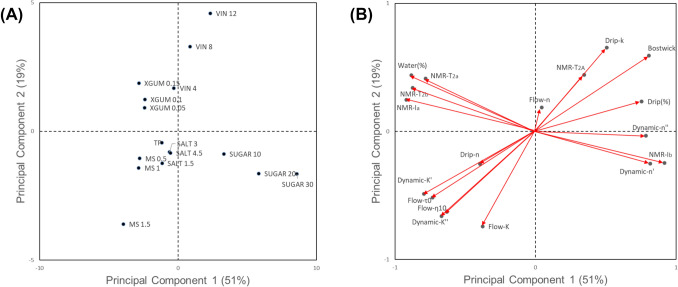
Principal components analysis of ingredients and physicochemical properties of model tomato ketchup; **(A)** score plot and **(B)** loading plot for two principal components (PC1 and PC2)

PCA was performed to elucidate the relationship between serum separation and water mobility by classifying the tomato samples in a two dimensional plane (Fig. 3). Based on the loading values, serum separation variables were mostly loaded on the first quadrant, whereas the samples with added XG and MS were loaded on the complete opposite side representing a little serum separation. This was consistent with the experimental results describing a significant decrease in the drip (%) value with the addition of XG and MS (Sec. 3.1). Principal component (PC) 1 loaded variables T_2a_, T_2b_, and I_a_ at negative side, whereas *k* and drip (%) at positive side which suggests the degree of water mobility and size of less mobile part may negatively affect serum separation. The VIN and samples were loaded along with PC 1 and located close to the serum separation quadrant. This implies that the increase in serum separation upon VIN addition was due to the intramolecular water mobility properties itself, and may be attributable to the increase in WC with the addition of VIN changed the mobility characteristics. In the PCA loading plot (Fig. 3b), the SU sample was positioned in the region associated with I_b_, which represents the size of the more mobile component. This indicates that the increased serum separation observed with SU addition is more closely related to the expansion of the mobile fraction, rather than to changes in overall proton mobility. That is, it can be presumed that the amount of water that can interact with solid contents to form a structure decreased due to the dissolution of a large amount of SU, and thus the mobile water molecule part in the sample changed.

### Effects of ingredients on rheological properties

#### Viscoelastic and steady-state flow properties

The viscoelastic and flow behavior variables of the TP samples are shown in Table 2. The storage and loss modulus at 1 Hz were expressed as K' and K" for representative values of modulus. The change in modulus (as a function of frequency) was represented as n′ and n″, respectively, indicating the dependence on frequency, and the results were compared with those for TP. These rheological properties are related to the structural strength of the samples and their serum stability (Gujral et al., 2002). For the viscoelastic properties, the storage modulus (G′) was higher than the loss modulus (G″) for all samples, indicating a gel-like structure. In general, the weaker the gel strength, the greater is the dependence of modulus on frequency (Patel et al., 2015). As can be seen in Table 2, XG increased, n′ and n″ decreased significantly, showing a viscoelastic characteristic independent of frequency. Notably, the n values also decreased significantly upon XG addition, suggesting enhanced elastic behavior across the frequency range. In the SU samples, G′ and G″ decreased markedly and n′ and n″ increased, indicating frequency-dependent viscous properties. As previously mentioned, the addition of SU to TP likely induced water release via osmotic pressure, leading to separation and making the texture more fluid-like. Both moduli of SA samples showed no significant difference according to the concentration, showing viscoelastic properties similar to those of TP. The VIN samples exhibited similar viscoelastic properties as the SU samples, but with reduced frequency dependence.

**Table 2:** Parameters of viscoelastic and flow behavior properties of tomato source samples added each ingredient

Samples^1^	(wt%)^2^	Viscoelastic behavior^3^					Flow behavior^4^				
		K' (Pa·s^n'^)	n'	K'' (Pa·s^n''^)	n''		τ_0H_ (Pa)	K_H_ (Pa·s^n^)	n_H_	η10(Pa·s)	Bostwick FL (mm)
TP		1526.9(46.2)^cd^	0.139(0.006)^c^	457.6(20.2)^abc^	0.279(0.005)^abc^		64.1 (0.7)^bc^	3.8 (0.1)^abc^	0.60 (0.03)^ab^	7.96 (0.02)^d^	19.0(1.4)^fg^
MS	0.5	1693.6(21.6)^bc^	0.141(0.009)^c^	492.5(19.3)^abc^	0.263(0.000)^bcd^		77.5 (7.4)^cd^	5.0 (0.8)^abc^	0.61 (0.04)^ab^	9.79 (0.60)^e^	16.0(0.7)^gh^
	1	1822.6(44.3)^ab^	0.140(0.002)^c^	500.4(10.5)^ab^	0.258(0.001)^bcde^		89.3 (4.4)^de^	3.5 (0.6)^abc^	0.74 (0.06)^b^	11.11 (0.20)^e^	12.5(0.7)^hi^
	1.5	2009.0(111.3)^a^	0.131(0.007)^cde^	519.5(13.8)^a^	0.254(0.002)^bcde^		100.0 (1.3)^e^	7.0 (1.8)^c^	0.67 (0.06)^ab^	13.57 (0.57)^f^	8.3(0.4)^i^
XG	0.05	1457.0(31.8)^cd^	0.118(0.002)^de^	413.7(12.7)^abcd^	0.236(0.002)^cde^		64.4 (3.1)^bc^	2.6 (0.8)^ab^	0.66 (0.05)^ab^	7.53 (0.09)^bcd^	24.3(3.2)^def^
	0.1	1302.0(24.1)^def^	0.117(0.000)^de^	379.7(14.3)^abcd^	0.212(0.002)^de^		56.7 (3.2)^ab^	3.2 (0.0)^abc^	0.63 (0.03)^ab^	6.92 (0.15)^abcd^	22.8(2.5)^ef^
	0.15	1279.3(7.5)^def^	0.114(0.001)^e^	360.5(5.3)^bcd^	0.201(0.002)^e^		56.0 (3.5)^ab^	2.4 (0.3)^ab^	0.70 (0.01)^ab^	6.79 (0.20)^abcd^	22.5(2.1)^f^
SU	10	1265.3(47.0)^def^	0.134(0.011)^cde^	370.2(1.3)^abcd^	0.285(0.004)^abc^		55.4 (1.4)^ab^	3.5 (1.2)^abc^	0.63 (0.06)^ab^	7.20 (0.13)^abcd^	23.8(1.1)^def^
	20	1033.0(79.2)^fg^	0.163(0.003)^ab^	344.3(23.3)^cd^	0.302(0.004)^ab^		49.0 (0.1)^ab^	3.1 (0.4)^ab^	0.64 (0.02)^ab^	6.40 (0.17)^ab^	29.0(1.4)^cd^
	30	985.8(46.9)^g^	0.175(0.002)^a^	343.2(22.3)^cd^	0.323(0.002)^a^		42.6 (2.0)^a^	3.2 (0.9)^abc^	0.67 (0.03)^ab^	5.96 (0.44)^a^	40.8(1.1)^a^
SA	1.5	1524.5(148.4)^cd^	0.137(0.006)^c^	513.8(136.3)^ab^	0.250(0.056)^cde^		61.2 (4.6)^bc^	5.3 (0.7)^abc^	0.53 (0.01)^ab^	7.90 (0.27)^d^	18.5(0.7)^fg^
	3	1338.8(72.8)^de^	0.137(0.002)^cd^	413.4(6.4)^abcd^	0.280(0.004)^abc^		59.4 (9.1)^abc^	5.8 (1.2)^bc^	0.51 (0.04)^a^	7.78 (0.58)^cd^	19.0(0.0)^fg^
	4.5	1456.0(73.6)^cde^	0.144(0.003)^c^	442.3(7.2)^abcd^	0.279(0.004)^abc^		62.0 (4.9)^bc^	4.1 (1.4)^abc^	0.58 (0.05)^ab^	7.76 (0.16)^bcd^	19.5(0.7)^fg^
VIN	4	1366.6(88.6)^efg^	0.148(0.001)^bc^	389.2(17.4)^abcd^	0.296(0.008)^ab^		62.2 (7.4)^bc^	3.5 (0.7)^abc^	0.60 (0.01)^ab^	7.55 (0.47)^bcd^	28.5(0.7)^cde^
	8	1202.8(0.3)^de^	0.142(0.008)^c^	351.8(25.4)^cd^	0.291(0.003)^ab^		53.2 (3.5)^ab^	2.8 (1.7)^ab^	0.66 (0.16)^ab^	6.49 (0.02)^abc^	32.8(0.4)^bc^
	12	1066.4(51.1)^fg^	0.142(0.002)^bc^	303.3(13.3)^d^	0.303(0.000)^ab^		49.0 (5.2)^ab^	1.9 (0.2)^a^	0.69 (0.02)^ab^	5.87 (0.42)^a^	37.5(2.1)^ab^

^1^ The samples were named as an abbreviation of ingredient added to tomato paste (TP). *TP* tomato paste without added ingredients, *MS* modified starch, *XG* xanthan gum, *SU* sugar, *SA* salt, *VIN* vinegar

^2^ It refers to the concentration of added ingredients (wt%)

^3^ K’, storage modulus at 1 Hz; K’’, loss modulus at 1 Hz; n’, storage modulus slope to frequency; n’’, loss modulus slope to frequency

^4^ τ_0H_, yield stress; K_H_, consistency coefficient; n_H_, flow behavior index; η_10_, apparent viscosity at 10 s^−1^; Bostwick FL, flow Length

Values are given the mean followed by the standard deviation in parenthesis. The lowercase letters indicate significant difference among samples (*p* < 0.05)

The flow behavior was applied to the Herschel-Bulkley equation. The yield stress (τ_0H_) is the minimum stress required to initiate flow and related to the structure of the suspension, an indicator of the strength of the gel network (Ma and Barbosa-Cánovas, 1995). The consistency of tomato ketchup is determined by its ingredient components and manufacturing process. The τ_0H_ value increased with increasing MS concentration and decreased with increasing concentrations of SU, whereas there were no significant differences according to SA or VIN concentration. The flow behavior index (*n*_H_) indicating the degree to deviate from the properties of a Newtonian fluid as the distance from 1 was less than 1 for all samples, and the viscosity decreased as the shear rate increased, indicating shear thinning. Overall, ingredients addition resulted in changes in the consistency coefficient (*K*_H_) indicating the degree of resistance to flow. Although *K*_H_ increased with higher MS concentration and was lowest in the XG sample, the differences were not statistically significant. 

#### Bostwick consistency and apparent viscosity

The results of the Bostwick consistency (BC) and apparent viscosity (η_10_) at a shear rate of 10 s^−1^ according to the addition of each ingredient are also shown in Table 2. In general, the BC increases with decreasing Bostwick flow length (mm). The addition of MS resulted in a more significant decrease in flow length (8.3 mm at 1.5% MS) with increasing concentration. This suggests that MS conferred a higher flow resistance on TP, resulting in enhanced consistency. Conversely, the BC increased with increasing concentrations SU and VIN, leading to the formation of a weaker matrix, which may be attributed to phase separation or the high water content introduced during formulation. The addition of XG resulted in an overall increase in BC; however, the differences among concentrations were not statistically significant. This finding is inconsistent with several previous studies reporting that the addition of XG generally increases viscosity (Koocheki et al., 2009; Mirzaei et al., 2018). However, Park et al. (2018) observed that the addition of XG at low concentrations (< 0.5%) induced more viscous properties, which is consistent with the present study. This may be attributed to the enhanced mobility of water molecules. As shown in Fig. 2, both T_2a_ and T_2b_, which reflect overall molecular mobility, increased with the addition of XG, suggesting an increase in viscous characteristics. The apparent viscosity (η_10_) also showed the same tendencies as the BC, with MS increasing the viscosity, while XG, SU, SA, and VIN decreased it in a concentration-dependent manner. The addition of SU and VIN resulted in the greatest decreases in viscosity, followed by XG and SA. The apparent viscosity indicated a high correlation with the result of BC (*r* = − 0.834, *p* < 0.05), increasing the reliability of the consistency tendency of tomato samples (Fig. S2).

#### Relationship between serum separation and rheological properties

Figure 3 also shows the PCA plot displaying the relationships between rheological properties and serum separation. For PC 1, variables indicating viscoelastic or flow behavior properties (K′, K″,τ_0H_, and η_10_) were closely loaded in the opposite direction from serum separation variables (*k* and drip [%]) (Fig. 3b). These results suggest that the gel-like structure in frequency-dependent samples may be easily disrupted, leading to increased serum separation. In general, the yield stress or viscosity of the tomato samples showed an inverse relationship with serum separation, where high serum separation was associated with low rheological values. In the PCA results, MS and XG samples were positioned on the same side as viscosity-related variables and on the opposite side of serum separation variables, indicating that their addition contributed to both higher rheological values and enhanced serum separation stability. This supports the findings in Sects. 3.1 and 3.3.2, which showed that MS and XG improved serum separation stability. These results align with the general observation that higher viscosity is associated with better serum stability (Sahin and Ozdemir, 2004).

In conclusions, the effect of different ingredients such as SA, SU, VIN, XG, and MS on the physical properties and serum separation behavior of TP were systematically investigated. LF-NMR data revealed that the addition of VIN significantly increased water mobility, while SU produced unique NMR signals likely due to protons present in the sucrose structure. Interestingly, the addition of XG led to an increase in the bound water population and a decrease in the mobile fraction, while overall mobility decreased, contributing to reduced serum separation. In contrast, serum separation occurred rapidly with the addition of SU and VIN, whereas SA delayed the onset of separation. Multivariate analyses, including PCA and regression, demonstrated that serum separation rate (k) and extent (drip [%]) could be effectively predicted based on LF-NMR variables and other rheological parameters. These findings suggest that combining NMR-derived mobility data with physical measurements allows for accurate prediction of serum separation behavior depending on the type and concentration of added ingredients. Ultimately, this approach provides a useful framework for optimizing the formulation of tomato-based products to improve structural stability and minimize undesirable phase separation.

## Supplementary Information

Below is the link to the electronic supplementary material.

Supplementary file1 (DOCX 385 KB)
