# Supplementary material for: Ingredient-dependent water mobility and physicochemical properties of model tomato ketchup in relation to serum separation
Source: Food Sci Biotechnol. 2025 Jul 16;34(14):3283–93. doi: 10.1007/s10068-025-01947-3 (PMC12408873; doi:10.1007/s10068-025-01947-3)
Supplement: Supplementary file 1 — Supplementary file1 (DOCX 385 KB) [file 10068_2025_1947_MOESM1_ESM.docx]

**
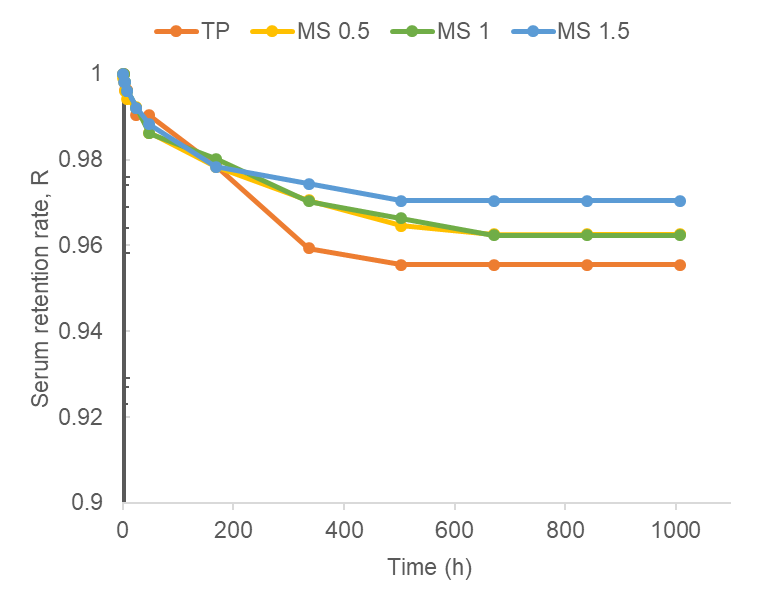
**
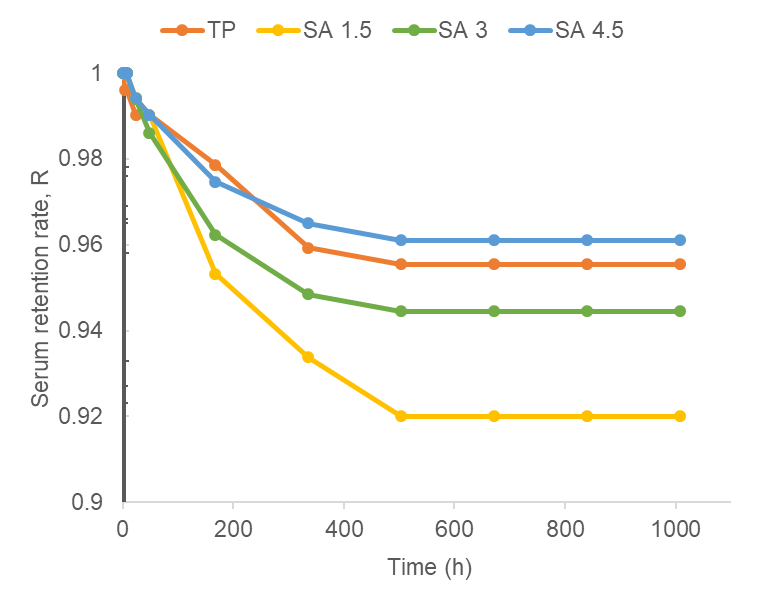

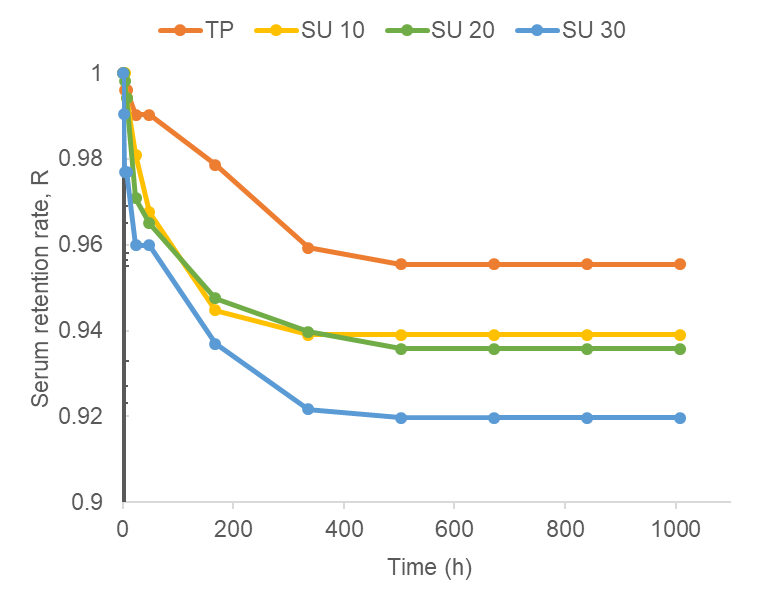
**
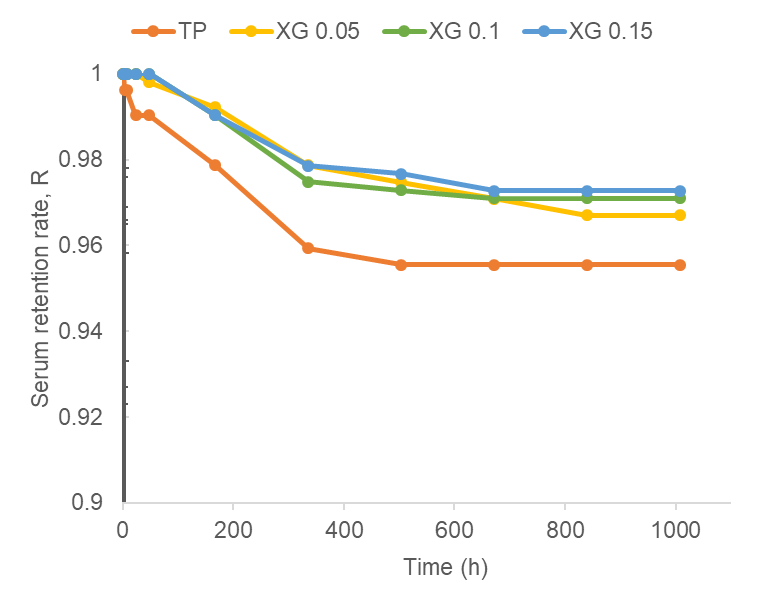
**


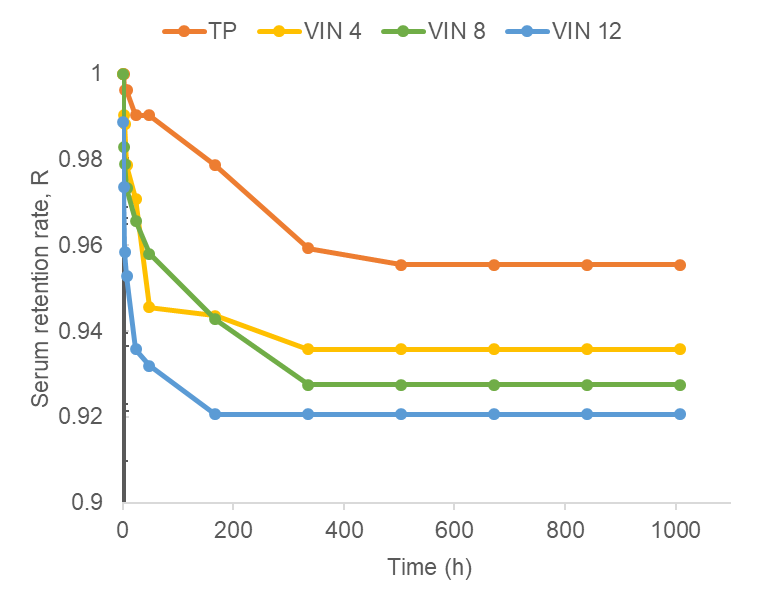


**Fig. S1.** The serum retention rate (R) during storage of model tomato ketchup with different supplemented ingredients. The numbers following sample abbreviations indicate the concentration (wt%) of supplemented ingredients to the tomato paste. TP = tomato paste; MS = TP + modified starch; XG = TP + xanthan gum; SU = TP + sugar; SA = TP + salt; VIN =TP + vinegar.


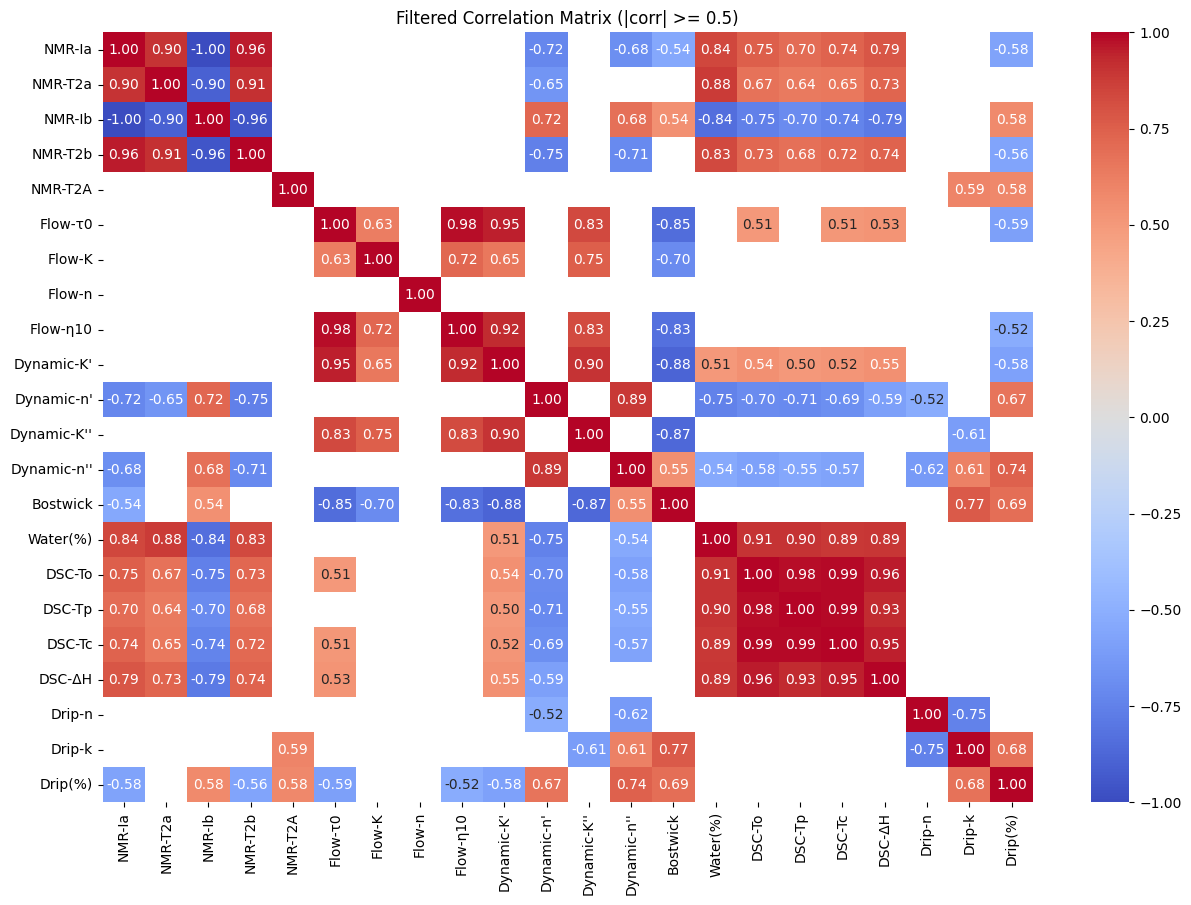


**Fig. S2. Correlation matrix for measured physicochemical properties of model tomato ketchup samples. Absolute values of correlation coefficient greater than 0.5 are presented.**
